# Supplementary material for: Are carbon emissions trading and green financial instruments synergistic? -Comprehensive quantitative research based on content analysis
Source: PLoS One. 2024 Mar 7;19(3):e0298601. doi: 10.1371/journal.pone.0298601 (PMC10919601; doi:10.1371/journal.pone.0298601)
Supplement: S1 Data — (ZIP) [file pone.0298601.s001.zip › Data/地级市Z_F工作报告.zip!/╣π╢1⁄2/╒┐╜¡╩╨/└┤╫╘╣1⁄2╓┌║┼í╛┬φ┐╦╩2╛▌═°í┐_2015.docx]

各位代表：

　　现在，我代表市人民政府向大会报告政府工作，请予审议，并请市政协各位委员和其他列席同志提出意见。

　　2014年工作回顾

　　刚刚过去的一年，国内外经济形势复杂多变，经济下行压力贯穿全年。面对困难和挑战，全市人民在中共湛江市委的领导下，主动适应经济发展新常态，团结一心，奋发有为，湛江又收获了一个丰收年。

　　2014年是开放之年，湛江列入国家“一带一路”海上合作战略支点；保税物流中心（B型）获得国务院批准建设，湛江港区扩大开放获得国家批准；中国海洋经济博览会盛况空前，开放湛江阔步向前！

　　2014年是突破之年，固定资产投资突破1000亿元，外贸进出口总额突破60亿美元，港口吞吐量突破2亿吨，机场客流量突破100万人次，红土大地蓬勃发展、充满生机！

　　2014年是沸腾之年，湛江钢铁基地万人施工、厂房林立，历经30年蓄势待发，东海岛终于成为热火朝天的大工地，湛江人民的钢铁激情全面点燃！

　　2014年是蝶变之年，历经八年奋战，成功获得国家卫生城市的荣誉；开展交通秩序综合整治，完成15条主干道“白改黑”，市区面貌化蛹成蝶、焕然一新；空气质量全省第一，红嘴鸥翱翔湛江湾，市民自豪、各方点赞！

　　2014年是惠民之年，十大民心工程加快推进，十件民生实事全面完成，扶贫开发“双到”工作扎实有效，打响告别茅草房大会战，1909户风灾“全倒户”春节前可全部搬入新居！

　　2014年是防灾之年，建国以来全省最强台风“威马逊”、强台风“海鸥”正面袭击徐闻，重创湛江。全市人民科学防御，沉着应对，创造了人员“零死亡”的奇迹，取得了防御台风、灾后重建的重大胜利！

　　过去一年，面对自然灾害频发、新旧矛盾交织的形势，我们在市委领导下，在市人大、市政协监督支持下，认真贯彻党的十八大以来中央一系列决策部署和习近平总书记系列重要讲话精神，全面落实省委省政府振兴粤东西北发展战略，全力促改革、稳增长、调结构、惠民生、防风险，开创了各方面工作新局面。

　　一年来，我们主要抓了以下工作。

　　一是抓发展不动摇，经济运行稳中有进。坚持以重点项目、重点工作为突破口，着力优化结构、扩大投资，创新供给、提振消费，强化服务、稳定生产，全力保持经济持续发展。实现生产总值2258.7亿元，增长10%。规模以上工业总产值2246.6亿元、增长14.8%，增加值734.9亿元、增长12.1%。固定资产投资1020.8亿元，增长36.2%；社会消费品零售总额1162.1亿元，增长13.3%；外贸进出口总额63.2亿美元，增长14.6%。公共财政预算收入114.4亿元，来源于湛江的财政总收入564.2亿元。居民人均可支配收入15301.8元，增长10.9%。城镇新增就业7.7万人，农村劳动力转移就业8.3万人。

　　二是抓改革开放，市场活力进一步释放。确定启动239项改革，实施试点81项。同步推进商事登记和行政审批制度改革，取消调整行政审批事项189项，改革投资管理，落实结构性减税，新增市场主体3.3万户，其中私营企业4456户、个体工商户2.7万户；民间投资606.3亿元，增长25.1%；民营经济增加值1439.5亿元，增长13%。引入省航运集团注资6.7亿元控股湛江航运集团，基投集团、开发区新域公司等融资平台成功发行债券。主动融入21世纪海上丝绸之路建设，设立驻德国、斯里兰卡和柬埔寨经贸代表处，在“新丝路”国家新增投资5.5亿美元，增长18.6倍；实际利用外资1.5亿美元，增长14%。成功举办中国国际水产博览会，雷州乌石对台小额贸易点获得批准，徐闻海安新港口岸开放通过验收。区域合作不断深化，制定《推进湛江与广西北部湾城市群一体化工作方案》，环北部湾“三关四港”七方协同发展顺利推进，积极参与北部湾和粤西城市群交流合作；与青岛市建立战略合作关系。

　　三是抓产业振兴，发展后劲进一步提升。重大工业项目加快建设。实施重点项目123个，新开工26个，建成21个，完成投资422.9亿元，增长14.2%。湛江钢铁基地完成投资116亿元，高炉、炼钢、连铸、热轧、冷轧等九大主体工程建设进展顺利，自备电厂1号机组并网调试。中科炼化项目具备全面开工条件。晨鸣一期运行良好，二期建成投产，全厂完成产值95亿元。中国纸业200万吨高档涂布纸项目动工。冠豪高新特种纸一期、结晶果糖等项目建成投产。遂溪中能酒精、廉江丰诚水泥加快建设。大唐雷州火电获得核准。京信电力前期工作基本完成，京能徐闻火电、华电廉江电厂前期工作加快。产业园区建设深入推进。“三讲三评”签约项目70个，计划投资178.8亿元。产业园区获得省产业政策性扶持资金6.95亿元，园区工业总产值突破1000亿元，完成固定资产投资315.4亿元。湛江、廉江两个产业转移园区全省目标考核优秀。湛江产业转移园完成投资133.5亿元，建成项目6个，工业投资任务完成率296.7%，排名全省第一。奋勇高新区6家企业投产。廉江产业转移园5个服务平台、12个项目和吴川产业转移园5个项目建成。海田物流产业园完成投资33亿元，建成15个专业市场。服务业发展迈上新台阶。全市港口吞吐量2.02亿吨，增长12.4%。机场客流量101.2万人次，增长43.6%。引进光大、兴业银行等7家金融机构；设立1718个农村金融服务站点，实现普惠金融服务行政村全覆盖；设立政银企互保金融贷款风险补偿金；金融机构存款余额2430.3亿元，存贷比56.5%；金融业增加值40亿元，增长26.3%。建成一批商贸流通项目，城市原点广场、万达广场一期基本建成。完成旅游投资22.3亿元，霞山法式风情街建成开放，徐闻荣获“中国长寿之乡”称号。“湛蓝的海、湛蓝的天”旅游品牌影响扩大，过夜游客1500万人次，旅游总收入201.8亿元，增长29.8%。出台促进“三旧”改造和房地产健康发展系列政策，成功举办“沈阳·发现湛江”城市推介会，多策并举增强房地产市场信心。现代农业持续发展。新增农业龙头企业14家，建成高标准基本农田73万亩，廉江、徐闻成为国家现代农业示范区。编制完成雷州东西洋现代粮食产业示范区总体规划，正大（湛江）现代农业种猪项目进入生产期，北部湾农产品流通综合示范园建成5万亩基地。政策性水稻、甘蔗保险参保率达到88.9%和91%。有序流转土地120万亩，完成土地承包经营权确权登记颁证廉江试点。启动渔船“小改大、木改钢”，新建造200吨以上大型钢质渔船30艘。修复海堤等水毁工程38宗，完成小型病险水库除险加固工程96宗，新建恢复小型水源工程1026处，新增有效灌溉面积14.9万亩。科技创新成效明显。“南方海谷”列为全省创新驱动发展重点建设平台。组建科技金融服务中心，建成市科技企业孵化器和大学生创业孵化示范基地，入孵科技企业31家、入驻创业团队45个；引进创新团队3个，新增高技能人才2455人；专利申请量增长40.7%，增幅全省第一。

　　四是抓扩容提质，新型城镇化进一步加快。交通基础设施建设有新起色。编制城市综合交通等规划，完成交通建设投资53.2亿元，东海岛铁路和云湛高速湛江先行段开工，海湾大桥西连接线二期建成。合湛高铁用地预审获批。湛江国际机场迁建选址获国家民航总局批准。湛江港霞山港区30万吨级散货码头完成重载调试。水上巴士一期3条航线开通在即。完成电网改造投资20亿元。海东开发有新亮点。海东新区管委会挂牌成立，新区完成征地1809亩，回收滩涂4905亩；动工27个项目、14个项目建成，完成投资43.7亿元；海东快线加快建设，完成投资计划61%；成功争取9.3亿元省粤东西北振兴发展股权基金。海西提质有新进展。市区24个“三旧”改造项目加快推进，同步实施棚户区改造。外贸码头实现功能腾退。西澜湾渔人码头基础工程竣工，完成投资9.8亿元。新湖大道、海帆路建成通车，开工建设源珠路、振兴路等9条市政道路；广州湾大道建设进展顺利，完成投资6300万元。建成50公里市区绿道网和一批小绿地、小游园。整治6个“水浸街”黑点，升级改造市区173条街道、15个路口，完成观海长廊、东海大堤防洪设施维修。生态保护有新提升。城市环境持续优化，PM2.5年均值达到国家二级标准，成为省循环经济试点市，生态文明区城镇村五年行动扎实推进。完成赤坎水库和南柳河等市区8条河渠截污整治，饮用水源和近岸海域水质达标率100%。签署九洲江流域跨界水环境保护及水污染联防联治协议，整治后的鹤地水库水质达到近五年来最好水平。鉴江中下游纳入全国重点入海河流治理工程。开发区平乐再生水厂等12个城镇、园区污水处理厂建成运行；冯村垃圾焚烧发电厂如期推进；吴川生活垃圾处理场建成使用，启动建设廉江垃圾焚烧发电厂。完成10个工业废水治理减排项目，关停6个重污染结构减排项目。开展排污权交易试点。淘汰“黄标车”2.5万辆，淘汰10蒸吨以下燃煤锅炉72台，超额完成淘汰任务。新增纯电动公交车15辆；建成首批市区公共自行车租赁点272个，投放自行车5000辆。复耕复绿违法违规占用土地1217亩，完成南三岛千亩防护林、徐闻曲界万亩“热带雨林”首期等生态修复工程，更新造林20.5万亩。

　　五是抓惠民工程，社会民生进一步改善。市财政社会民生投入203亿元，增支25.5亿元，占公共财政支出70.6%。民生保障持续加强。支持创业3086人，扶持就业困难人员再就业1729人。企业职工养老保险待遇连续十年按10%左右比例提升，建立全市统一的城乡居民基本养老保险制度，基本实现社会保障卡户籍人口全覆盖。城乡居民基本医疗保险参保率98.8%，异地就医住院费用即时结算定点医院增至8家。完成城乡低保提标、五保达标工作，市级养老服务中心动工。新建平价商店30家、药店37家。开工建设保障房3154套、23万平方米，基本建成2331套、13万平方米。动工改造茅草房10026户，竣工5466户。完成200条自然村“村村通”自来水工程。扶贫开发“双到”投入资金13.6亿元，建成项目4691个。社会事业持续进步。50条基本公共服务均等化试点村建设持续推进。创建3个义务教育基本均衡县、5个教育强县（市、区）、32个强镇（街）和340个特色文化校园，改造提升农村薄弱义务教育学校213所、普通高中87所；新建改建特殊教育学校5所，建成2所民办实验中学和湛江卫校、机电学校新校区一期主体工程；省二类城市语言文字工作通过评估；统筹城乡教育资源，1109名城市教师到农村支教，免费培训农村教师4.2万人，全面落实农村边远地区教师岗位津贴。启动“诊疗一卡通”试点，完成中心人民医院新院主体工程；在全省率先推行食品药品网格化监管，“阳光厨房”经验在全国推广；成功防控H7N9禽流感和登革热等重大疫情；27名专家远赴赤道几内亚执行援非医疗任务，协助防控埃博拉疫情。落实“单独两孩”政策，人口自然增长率6.43‰。基本建成奥体中心主场馆，水上运动中心加快建设；湛江籍运动员参加仁川亚运会取得3金2银1铜好成绩。特色文化精彩纷呈，大型雷剧《岭南圣母》公演；建成9个镇（街）文化站、367个村（居）文化室；吴川（飘色、泥塑）上榜“中国民间文化艺术之乡”。市档案馆新馆启用。双拥共建、国防教育进一步加强，军民融合发展大好局面更加巩固。海关、检验检疫、海事、边检等口岸单位深化关检合作，支持地方发展。外事侨务工作获外交部“因公护照管理服务贡献奖”。投入1820万元购置的云梯消防车正式启用。审计、统计、粮食、老区、海防打私、人防、气象、方志、残疾人、防震减灾、民族宗教、妇女儿童等工作取得新成绩。社会大局持续稳定。加强综治、信访、维稳和调处工作，开展“社会矛盾化解年”活动，平安湛江建设全面推进，创新社会治理经验被中央综治委推广。集中整治治安突出问题，新装治安视频监控镜头11000多个，开展"6+3"专项行动，有效遏制涉枪、涉赌、涉毒、涉黑、暴力阻工以及“两抢一盗”犯罪。依法引入信达公司重整嘉粤集团。连续六年未发生重特大安全事故。开展“一村（社区）一法律顾问”试点，成为全国“六五”普法中期先进城市。

　　六是抓作风转变，政府建设进一步加强。扎实开展群众路线教育实践活动，严格落实中央“八项规定”，全面压缩会议、文件和评比表彰活动，“三公”经费下降27.9%。主动接受人大依法监督和政协民主监督，办理人大代表建议、政协委员提案216件。在全省率先建立政府规范性文件公示备案系统。1784个村（居）开通网上办事点，120个镇（街）建成网上办事服务站；市政府行政服务中心受理办件18万件，办理率100%。完善局长轮流接听群众来电制度，247名市直部门领导接听市民来电；12345市民服务热线接听来电27.3万宗，办结率94.9%，群众满意率90%。及时公开政务信息，举办市政府新闻发布会14场、在线访谈17次；市政府门户网站获得中国特色政府网站“在线服务创新奖”，新版政府英文官网开通，微博、微信成为政务公开新平台。

　　各位代表，过去一年，湛江干成了一批大事、实事，成绩来之不易。这归功于市委科学决策，归功于市人大、市政协有效监督，归功于人大代表、政协委员大力支持，归功于驻湛部队、武警官兵、公安干警不辱使命，归功于中央、省驻湛单位和民主党派、工商联以及社会各界人士团结协作，归功于全市干部群众辛勤付出，我谨代表市人民政府，表示衷心的感谢，致以崇高的敬意！

　　一年来，政府工作还存在许多不尽人意的地方，距离人民群众的期待还有较大差距。主要是：经济下行压力增大，GDP等主要经济指标未能达到全年预期；财政收入增速下降；工业增长乏力，中科炼化等重点工业项目推进不快；消费热点不多，房地产市场不振；生产性服务业层次不高；创新驱动能力不足、氛围不浓，新兴产业发展缓慢；有的改革措施落实不到位，依法行政能力有待加强等等。对此，我们要高度重视，坚决克服。

　　2015年工作部署

　　今年市政府工作的总体要求：深入贯彻党的十八大、十八届三中、四中全会和习近平总书记系列重要讲话精神，认真落实省委省政府振兴粤东西北发展战略，牢牢把握发展第一要务，坚持稳中求进工作总基调，主动适应经济发展新常态，按照市委十届七次全会决策部署，着力抓好陆海统筹、城乡统筹、创新驱动三件大事，着力践行海绵城市、循环城市、脚印城市“三个城市”建设理念，着力落实改革开放、生态建设、民生改善三项任务，奋力完成全年及“十二五”经济社会发展目标任务。

　　今年经济社会发展主要预期目标：生产总值增长10%左右，人均生产总值增长8%；工业增加值增长12.5%；固定资产投资增长16%；社会消费品零售总额增长13%；港口吞吐量增长10%；外贸进出口总额增长8%；一般公共预算收入增长10%，居民人均可支配收入增长8%；城镇登记失业率控制在3%以内，居民消费价格涨幅控制在3%左右；万元GDP能耗下降2.9%。

　　抓好今年工作，实现全年目标，关键在于清醒认识我国已经进入速度换档、结构优化、动力转换的经济发展新常态，清醒认识新常态背景下湛江发展的新内涵、新形势和新要求。适应新常态，实现新发展，我们面临新挑战，破解动力转换与瓶颈制约的“双重矛盾”，防范重大项目配套资金巨额投入与各类社会矛盾积累的“双重风险”，完成后发追赶与坚守生态底线的“双重任务”，实现城市发展与产业结构的“双重转型”，考验着我们的能力与智慧。我们迎来新机遇，新常态下涌现的新技术、新业态、新动力为我们拓展了新空间，增添了新主体，提升了新后劲。我们具有新底气，以湛江钢铁基地为代表，蓬勃兴起的临港大工业有利于推进新型工业化；以海东开发为代表，人气旺盛的“一湾两岸”中心城区、初具规模的特色城镇链，有利于推进新型城镇化；以保税物流中心建设为代表，扩大开放的南方深水大港，有利于全面融入国家“一带一路”大战略。我们探索新路径，围绕推动发展、加快发展这一主题，更加重视陆海统筹，科学利用海洋资源，加快推进港口群建设；更加重视城乡统筹，全面推进基本公共服务均等化；更加重视全面创新，驱动“湛江号”这列动车在发展的轨道上行稳致远。我们应有新作为，新常态下发展依然是第一要务，湛江是欠发达地区，我们必须在保证发展质量、保护生态环境的前提下，保持合理的较快的发展速度，与全省同步率先全面建成小康社会。

　　适应新常态，实现新发展，今年主要抓好以下工作。

　　一、更大力度抓改革开放，充分释放发展活力

　　全面深化改革激发活力。坚持以处理政府与市场的关系为核心，着力打破捆住群众手脚、束缚市场活力和社会创造力的不合理体制。转变政府职能，建立政府权责清单和负面清单，理清事权边界和职责分工，严禁法外设定权力，以政府权力的“减法”换取市场活力的“加法”。深化行政审批制度改革，继续取消、下放行政审批事项；坚持放管结合，完善商事登记制度，加强事中事后监管；完善镇（街）行政管理体系。推进事业单位分类改革。完成公务用车制度改革。完善全口径预算体系，促进财权事权相匹配。转变国资监管方式，深化国有企业改革，发展混合所有制经济。深化价格改革，加强价格监管，完善价格服务。继续推进海域使用权“直通车”改革试点。深化银政银企合作，引进更多金融机构，完善地方金融服务体系；发展资本市场，扩大直接融资比例；推动农信社改制，争取雷州市农信社改制为农村商业银行；促进融资担保行业健康发展，用好中小微企业发展资金池，力促金融活水更多流向“三农”，流向中小微企业，流向实体经济。

　　深度融合建设“新丝路”。抢抓湛江列入国家“一带一路”海上合作战略支点的机遇，制定实施战略支点城市建设规划，深化务实合作，扩大对外开放，着力构建开放型经济。继续办好中国海洋经济博览会，扩大与东盟等“新丝路”沿线国家和地区经贸文化交流合作，开辟更多国际集装箱班轮航线；支持湛江农垦实施印尼剑麻、斯里兰卡甘蔗压榨等项目，开放雷州乌石对台小额贸易点和吴川进出境货运车辆检查场。主动融入两广经济一体化，积极参与北部湾城市群一体化，共促环北部湾港口协同发展，共建粤桂北部湾经济合作区；参与“湛茂阳”、珠三角、港澳台以及青岛市、大西南等区域合作。拓展跨境电子商务，巩固欧美传统市场，开拓金砖国家新兴市场，培育新的外贸增长点。完善政务中心等口岸查验设施，推广一次申报、一次查验、一次放行“三个一”模式，加快区域通关一体化，提升通关便利化水平。

　　二、更大力度抓陆海统筹，发展壮大蓝色产业

　　推进陆海统筹，必须充分发挥海洋优势，实施港口带动战略，促进县域经济发展。要落实好张德江委员长关于加快湛江港口建设的重要指示精神，大力发展临港工业，优化港口服务业，打造半岛港口群，扩大港口辐射范围，统筹开发陆上国土与海上国土，加快实现蓝色崛起。

　　推动工业进岛，建设临港产业高地。安排重点项目135个，新开工项目33个、建成项目22个，完成年度投资453亿元。坚持把服务和保障湛江钢铁基地建设作为今年经济工作的头等大事，推动完成投资160亿元，确保钢前系统9月建成投产。推动中科炼化项目尽快完成工程设计优化方案，争取尽快全面动工。加快建设钢铁配套园区，启动石化园区路网工程，引进更多配套项目，集聚发展钢铁石化产业。推动实施中国纸业200万吨高档涂布纸、鹏尊能源等项目，力争京信电力等项目开工。建成东海岛疏港公路、岛东大道和500千伏输变电项目，推进红星水库扩容工程前期工作。

　　推动企业入园，壮大县域经济。深入开展“三讲三评”，完善县域经济考核激励机制，支持县（市、区）创造条件加快发展。坚持“抓大不放小”，依托园区吸引更多项目落地生根，壮大县域工业，结出“星月齐辉”的产业硕果。坚持基础先行、管网先建，完善园区配套设施；创新投融资模式，鼓励园区引进工业地产建设标准厂房，强化企业、产业招商，提升招商质量和效果。坚持项目第一，支持晨鸣二期达产达效，三期如期投产，四期加快前期工作，同步建设太平森工产业园；动工建设大唐雷州火电，支持遂溪中能酒精、廉江丰诚水泥、中海油80万吨重交沥青和华电徐闻华海、粤电徐闻曲界、石板岭以及雷州红心楼4个风电场等项目建成投产；推进京能徐闻火电、国电吴川能源、华电廉江电厂等项目前期工作。加快奋勇高新区开发建设，完善5400亩启动区基础设施，打造粤西食品医药集聚群基地，建设军工产业园和中国-印尼合作以及新能源产业园。支持湛江临港工业园和廉江、吴川产业转移园扩能增效，推动海田物流产业园转型升级，加快建设坡头龙头、遂溪岭北、徐闻下桥等新兴产业园区，鼓励申报省级产业转移园。规划建设吴川空港经济区。

　　推动交通联网，完善“三环四通”格局。动工建设东海岛至雷州高速、湛徐高速徐闻港支线以及合湛高铁，加快建设云湛高速湛江段和东海岛铁路，推进黎湛铁路电气化改造。坚持沿海、带港、靠镇，完善环半岛公路规划。完成湛江国际机场前期工作，力争年内动工建设，规划建设吴川至湛江环城高速东段的沿海高速公路以及汕湛高速吴川支线（机场快线）。推进环城高速南坡段、玉湛高速等项目前期工作。按照“迎国检”要求，完成国、省道改造550公里，全面实现湛江1136.9公里的国、省道达标升级；完成100公里县道大修和550公里乡、村道改造，加强公路养护，全面提升路容路貌，努力让群众不仅有路走，还要有好路走。

　　推动港口成群，提升南方大港地位。着力改变港口建设湾内湾外“一热一冷”现状，严控湾内填海造地，支持湾外有序用海，规划建设雷州半岛港口群。优化发展湾内港口，加快建设湛江港霞山、宝满、东海岛港区，推进湛江港30万吨级航道改扩建；建成保税物流中心（B型）并封关验收，构建多层次保税物流体系；加快发展以集装箱运输为代表的现代港口物流业，建设华南大宗商品交易中心，开拓内陆无水港，扩大港口货源。加快开发湾外港口资源，坚持政府主导、市场运作，发挥湛江港集团龙头作用，统筹推进半岛港口群建设，实施“一县一港、一港一园”县域发展带动战略。年内开工建设徐闻南山港；与大唐集团合作，加快建设雷州乌石港；与华电集团合作，加快建设廉江龙头沙港；结合湛江电厂搬迁，规划建设遂溪草潭港；规划建设吴川吴阳港，完成无水港口岸验收，并启动运作。依托港口开发，建设雷州、徐闻、遂溪、廉江和吴川临港产业园，形成港口带动、港园互动、海陆联动的县域经济发展新格局。

　　推动渔业转型，加快渔船渔港渔镇转型升级。支持在湛江湾外建设深水网箱集聚区，引导滩涂养殖、浅湾养殖有序退出，加快渔业转型升级。鼓励造大船、出远海，实施渔船“小改大、木改钢”，更新建造200吨以上大型钢质渔船60艘。鼓励企业建设远洋渔业基地，支持渔民成立捕捞专业合作社。专项整治渔港“脏乱差”，每个县（市）建设一个示范渔港，抓紧建设龙王湾避风港。突出渔港风貌、渔家风情、渔民风采，规划建设特色渔镇，打造现代渔业示范区。

　　推动旅游靠湾，提升“五岛一湾”开发水平。实施滨海城市旅游规划，坚持把城市当景区精心策划、专心经营、用心推介。推进南三岛滨海旅游开发，落实缤纷欢乐海岸、休闲渔港和环岛公路等项目，打造中国南方冬休度假基地。完善金沙湾、渔港公园旅游设施，建设市区旅游标识示范点，启动法式风情街二期工程，实施渔人码头等13个重点项目，提升湛江湾旅游品质。支持徐闻打响“长寿之乡”品牌，发展古代海上丝绸之路“始发港”特色旅游，建设旅游综合改革示范县。规划建设南三岛邮轮停靠港，开发“新丝路”、北部湾邮轮航线，发展邮轮经济。

　　三、更大力度抓城乡统筹，加快推进新型城镇化

　　推进城乡统筹，必须从人口大市这一基本市情出发，坚持以人为本两手抓，一手抓“中心城市-县城-中心镇”城镇链建设，一手抓推进基本公共服务均等化，逐步缩小城乡差距，统筹城乡协调发展。

　　加快城镇链建设步伐。编制新型城镇化规划，完成城市总体规划修编审批工作。提速开发海东，完成新区起步区总体规划、控制性详规编制和土规调整。同步推进地面路网、地下管网、地上生态建设，落实综合管廊建设试点，加快土地储备和岸线整治。掀起路网建设热潮，确保6月底海东快线建成通车，加快沿海观光大道等10条道路建设。推进南粤金融大厦等项目，筹建电子商务城和动漫、高新技术产业孵化基地。升级改造起步区32条村庄，加快三柏西等3条村庄搬迁。提升发展海西，推进霞海港码头、调顺港区功能腾退，推动湛江化工厂、湛江电厂、海滨船厂环保迁建。抓紧抓好“三旧”改造，启动旧大天然片区改造。加快广州湾大道等市政道路建设。建成东盟公园，规划建设银帆公园。加快建设智慧城市，建成数字化城市管理中心，推进城市精细化管理。严厉查处违建行为，坚持党委撑腰、政府担责、市区联动、社会监督、严格问责，出台强有力措施，严厉打击违法建设，坚决铲除城市建设的“毒瘤”。做大县城和重点中心镇，编制县城及中心镇控制性规划，加快市政干道建设，适度扩大县城建成区面积，打造规模初具的中小城市。发展教育、科技、文化、卫生事业，提升中心镇基本公共服务水平。促进房地产健康发展，优化住房金融服务和公积金贷款方式，支持合理的住房消费。推动商品房户型“大转小”、毛坯房转精装房，探索发展产权式酒店公寓；突出冬休、度假、旅游三大题材，以北方城市为重点，面向全国加大宜居湛江形象宣传，让更多人共享湛蓝的海、湛蓝的天！

　　发展壮大现代农业。持之以恒强农业、富农村、惠农民，提升现代农业发展水平。平稳推进农村土地征收、集体经营性建设用地入市和宅基地制度改革，规范农村土地流转，开展农村集体“三资”清理工作。全面开展农村土地承包经营权确权登记颁证。编制实施廉江、徐闻国家现代农业示范区规划，启动建设雷州东西洋现代粮食产业示范区，支持湛江农垦建成重要农产品生产基地和现代农业的示范带动力量。落实粮食安全政府主体责任，完善粮食仓储物流设施。建成正大（湛江）现代农业肉鸡产业链一期。推广种植热带特色水果蔬菜，发展热带农产品精深加工业，建设直供港澳的果蔬种植加工基地。开展香蕉、玉米和涉农小额信贷保险。培育冷链物流龙头企业，强化冷链技术支撑，壮大冷链物流产业。健全农产品质量和农村饮水安全监管检测体系。启动南三岛省级新农村示范片工程。完善鉴江供水枢纽工程配套设施，确保3月向湛江钢铁基地安全供水。完成青年运河节水一期主体工程；处理好“水缸”与“米缸”的关系，推动鹤地水库水源保护与水库移民生产生活和谐发展。启动合流水库改扩建和甘村水库整治。修复一般水毁工程276宗，完成滨湖堤岸和7宗、70公里中小河流整治。

　　推进农村基本公共服务均等化。坚持政府为引领、农民作主体、多方齐参与，总结提升50条基本公共服务均等化试点村建设经验，扩大试点范围。注重从村庄、饮水、集市、教育、医疗、文体、生态、社保、法律援助和平台建设10个方面着手，健全农村基本公共服务体系。突出抓好村级基本公共服务中心建设，完善村级公共服务功能。培育壮大村级经济，努力消除集体经济“空壳村”。坚决落实农村生活垃圾集中整治方案，建成“一县一场”、“一镇一站”、“一村一点”设施，构建“户集、村收、镇运、县处理”体系，积极探索农村生活垃圾就近处理的途径与方法，全面扭转农村生活垃圾处理落后局面。推广“生物处理-中水灌溉”模式，鼓励有条件的农村建设小型循环污水处理厂。

　　推进“三地”务实合作发展。推进湛江新型城镇化，需要发挥驻军、高校、农垦三大优势，提升军地、校地、垦地“三地”合作水平。军地合作重在巩固军政军民团结，坚持在解决实际问题中增进感情，加强国防教育，推动军民深度融合发展，再创国家和省级双拥模范城。校地合作重在推进实施创新驱动发展战略，加快政产学研用一体发展，支持广东海洋大学、广东医学院、岭南师范学院和省部科研院所融入地方、服务湛江，增强区域创新发展能力。垦地合作重在推进城镇化、工业化，支持湖光农场建设垦地合作示范区，推动农场与毗邻乡镇融合发展，打造镇场一体化区域小城镇。

　　四、更大力度抓创新驱动，打造发展新引擎

　　推进创新驱动，首先要认清形势，提升能力，不能再输在起跑线上。资源瓶颈与环境约束带来的发展压力，要求我们不能再单纯依靠要素投入；以钢铁为代表的现代临港工业带来的发展机遇，要求我们营造开放的国际化的创新环境；以青年为代表的千万创业者，要求我们加快打造创新平台。我们必须把创新驱动作为总抓手、总支撑，统筹推进技术、产品、市场、组织和管理创新，力促创新成为驱动发展的新引擎，争当粤东西北创新驱动发展排头兵。

　　加快培育战略性新兴产业。创新科技管理体制，破除科研对接产业的体制性障碍，培育发展工业机器人、海洋装备、冷链物流、信息消费、生物制药、循环经济等新兴产业。扩大信息消费需求。加快完善公共设施服务网络、WiFi等信息基础设施，推进电信网、广播电视网、互联网“三网融合”，发展数字出版、互动多媒体、移动新媒体。申报建设国家信息消费示范市。推动电商与实体经济融合发展，开拓电子商务空间，支持企业在线经营，实施湛货网上行计划；培育城市社区和农产品电子商务，建设农业物联网应用示范园区。完善人才培育体系，支持“校企双制”培养技能人才，引进高层次人才，壮大创新型人才队伍。依托企业主体，发展其他新兴产业。

　　加快发展生产性服务业。培育发展文化创意、工业设计等新型服务业，支持发展科技、金融、绿色消费等新型服务业态，扶持发展小型化、智能化、专业化产业组织。依托中国海博会和湛江海洋周，举办珍珠、游艇、水产加工、海洋旅游、农产品等专业会展，壮大会展经济。支持发展服务贸易和服务外包，鼓励企业拓展离岸、在岸服务外包业务，建立与国际接轨的生产性服务业体系。加快建设市区、东海岛和县城中央商务区，推动万达广场、城市原点广场等城市综合体加快建设。完善城市配送物流体系，发展第三方物流，动工建设北部湾农产品流通综合示范园二期和湛江国际建材家居博览中心，建成湛江商贸物流城一期和霞山、赤坎小商品批发城。

　　加快推动技术改造和管理创新。推进信息化与工业化融合发展，普及数字化技术设备和智能控制系统。支持传统产业加大技改投入、转型升级，推动制糖企业技术更新，发展制糖精深加工；支持建设小家电研发中心，推动家电企业合作重组；支持家具、食品、纺织、羽绒、珍珠、烟草等产业改造升级，打造特色产业集群。支持企业加快管理创新，按照“个体工商户-中小微企业-规模以上企业-股份制企业-上市公司”的成长路径，推动“个转企、企转规、规转股”。

　　加快建设“南方海谷”。以湛江湾区为中心，推动创新链与产业链、资金链“三链”融合，建设“南方海谷”，是我市实施创新驱动发展战略的重要载体和平台。坚持海洋科技与海洋产业无缝对接、海洋科研与海洋生活有机结合；坚持更高水平发挥海洋资源优势，加快海洋产业提档升级；坚持服务海洋强国战略、服务广东海洋科技升级、服务东海岛工业新城和奋勇高新区建设，编制完成“南方海谷”总体和核心区规划，深化校地、院地融合发展，打造创新联盟，组建管理机构，招募运营团队，落实核心区选址，加快建设霞山智慧产业城启动区，打造“科研+市场+生活”的创新之园。

　　各位代表，经济发展进入新常态，创新正在成为经济提质增效、健康发展的核心动力。我们要以建设“南方海谷”为引领，坚定实施创新驱动发展战略，尽快形成大众创业、万众创新的生动局面，走出科学发展、持续发展的新路！

　　五、更大力度抓生态文明，以人为本推动城市转型发展

　　围绕建设生态型海湾城市目标，按照“车行要畅顺、慢行要宜人、水行要生态、风行要流通、物行要循环”的要求，把海绵城市、循环城市和脚印城市“三个城市”理念贯穿城市规划、建设、管理各环节，推动城市转型，带动产业调整，实现产城融合、升级发展。

　　建设海绵城市。坚持系统治水、科学治水、依法治水，优化城市水环境，建设自然积存、自然渗透、自然净化的海绵城市。注重在市政建设中减少水泥地，减少不透水的硬化铺装，减少占用涵养水源的林地、草地、湿地。基本完成市区主要河渠环境综合整治，联片整治城区河渠及上游农村污水，把东山垌建成重要生态湿地公园；构建完整的生物链、食物链和清水结构，把绿塘河建成自然河渠、生态廊道；改造旧城区排水系统，整治市区10个“水浸街”黑点。综合整治海湾沿线污染源，基本建成赤坎水质净化厂三期，动工建设海东新区污水处理厂，推进建设屋山污水处理厂。

　　建设循环城市。强化节能减排目标考核，落实环保“三同时”制度。完善重点工业企业脱硝、脱硫设施，扩大主城区“黄标车”限行范围，实施机动车环保标志管理，深入开展大气污染防治。秉承资源再生、综合利用，加快发展东海岛钢铁石化循环经济，创建国家循环经济示范市。注重从源头减少废弃物，完善城市生活垃圾处理体系，引导废品回收行业规范发展，用市场力量推动垃圾分类回收；建成冯村垃圾焚烧发电厂，加快建设医疗垃圾处理厂和廉江垃圾焚烧发电厂。编制林业生态建设规划，推进新一轮绿化大行动，更新造林20万亩；推动国有林场向生态公益林场转制，营建生态林4万亩。

　　建设脚印城市。严格划定城市生态控制线，推进编制脚印城市规划设计管理与规定标准等“八大行动”。坚持“公交优先”，编制实施城市公共交通体系规划，开辟市区主次干道公交专用车道，扩大新能源公交车应用，增开公交线路，建设公交首末站场，新增更新公交车346辆；完成城市轨道交通规划，发展大运量快速公交；规范合标电动车管理，加快建设市区自行车、电动车廊道，完善公共自行车租赁系统，打造便捷、高效、准点的公交都市。坚持“行人优先”，维护行人路权，增加人行道、绿道；完善人行天桥、地下通道等过街专用通道设施，建设“赤坎‘三民’路-金沙湾-霞山观海长廊-法式风情街-渔人码头”城市慢行景观廊道。坚持历史传承与现代元素有机结合，改造赤坎“三民”、霞山逸仙两大片区，打造“生活街道”、“活力街区”。加强交通需求管理，规范车辆停放，专项整治城市临街退缩公共空间；打通市区“断头路”，强化社区开放通达，推动城市道路微循环。

　　各位代表，新型城镇化不只是汽车机动化，小康社会不只是汽车社会。城市公共服务的中心始终是人，是让居住在城市的人们生活更美好。我们倡导广大市民少一点汽车生活，多一些绿色出行，步行出街购物，骑车公务上班，公交会友访亲，用双脚接地气，走出低碳，走出绿色，走出健康！

　　六、更大力度抓民生保障，努力改善人民生活

　　带着感情落实民生保障。坚持和谐发展，努力使城乡困难群众生活有希望，奋斗有回报。坚持就业为本、创业优先，完善就业保障服务体系；发挥创业孵化基地作用，营造宽松包容氛围，焕发自由创造激情，以创业带动就业。深化收入分配制度改革，完善薪酬制度，增加城乡居民收入。落实县（市）低保重新申请审核工作，推进异地务工人员基础服务与基本保障“底线均等”，编织更加牢固、更加精准的社会保障网。落实物价上涨与低保生活补贴联动机制，有效保障“两低群众”基本生活。改革机关事业单位养老保险制度，推进企业职工全员足额参加社会保险。落实带薪休假制度，鼓励分段休假。完善医保“湛江模式”，推动基本医保范围由大病向小病延伸，将儿童急性白血病等6种疾病纳入城乡儿童重大疾病保障范围；推进城乡医保门诊统筹，城乡医保二类医院住院报销比例提高到70%。坚持兜底救急，落实临时救助制度；抓好扶贫开发“双到”工作，实现250条重点帮扶村、16.4万贫困群众稳定脱贫。

　　办好十件民生实事：提高城乡免费义务教育生均公用经费补助标准，小学从950元提高到1150元，初中从1550元提高到1950元；城乡居民低保标准每人每月分别从370元、230元提高到410元和260元；城乡居民养老保险基础养老金每人每月从80元提高到100元；职工医保特定门诊病种范围由16种扩大到23种；解决68个社区办公用房不足问题；建立80岁以上老人普惠型高龄津贴制度；新装市区治安视频一类镜头1600个；开工建设保障房7112套、建成2884套；建设移民新村85条；解决200条自然村、20万人饮水安全问题。

　　推进十大民心工程：打好告别茅草房大会战，基本完成茅草房改造；完成中心人民医院迁建，推动市第一中医院、市妇幼保健院迁建扩建，加快建设广东医学院附属海东医院；动工建设湛江文化中心；推进残疾人康复中心建设；建成市级养老服务中心；加快建设职教基地湛江幼师高等专科学校、湛江卫校新校区二期；完成湛江钢铁基地安置小区村民回迁；强化鹤地水库水污染综合整治和鉴江中下游治理，整治袂花江、小东江、三叉江水患；开工建设海堤21宗、建成5宗，加固金沙湾海堤，完成堤围达标100公里；深化生态文明区城镇村五年行动，推进“五城同创”，巩固“创卫”成果，落实“创模”工作，抓好“创文”活动，让文明成为城市基因，生态成为湛江名片！

　　带着使命发展社会事业。实施强师工程，提升基础教育办学水平，创建教育强市；落实学前教育三年行动计划，新建改建6所市区义务教育学校，加快建设岭南师范学院附中新校区，推动新建小区配建学校；建设3所特殊教育学校；推进中小学文体设施向社会开放。坚持以病人为中心，以基层为重点，以信息化为手段，完成县镇医院一体化改革，推动市管医院与社区卫生服务中心合作共建，强化基层全科医师全员培训，努力让群众花最少的钱，走最短的路，享受最好的医疗服务。加强计生工作，提升优生优育服务。推进文化惠民工程，构建现代公共文化服务体系；强化文化遗产和传统村落保护，争取湛江海上丝绸之路史迹点列入国家名单，全力申报世界物质文化遗产。实施城市雕塑工程。启动建设市档案馆二期。做好外事侨务、民族宗教、妇女儿童、海防打私、人防、气象、地震、方志等工作。

　　举全市之力办好第14届省运会。办好第14届省运会和第7届省残运会，推动群众性体育运动蓬勃发展。加快场馆建设，完善配套设施，确保32个比赛场馆如期交付使用；精心筹备开闭幕式，抓好竞赛组织和运动员训练，落实交通、医疗、安保等后勤保障和新闻宣传工作，举办一届“富有特色、平安祥和、精彩节俭”的体育盛事。

　　带着责任创新社会治理。完善社会治理服务体制，加快建设平安湛江。创新社会组织登记管理，扶持发展公益慈善等社会组织，加大购买社会组织公共服务力度，推动社会组织依法自治。推进依法信访，引导群众依法表达诉求、维护权益。加强矛盾纠纷调解，积极化解建筑领域、工厂企业劳资纠纷。强化铁腕治赌，强力推进“3+×”专项行动，严厉打击各类违法犯罪，构建立体化社会治安防控体系。完善公共法律服务体系，落实“一村（社区）一法律顾问”制度，提高公民法治意识，加快建设法治社会。坚决治理餐桌污染,“零容忍”打击食品药品安全违法犯罪行为。提升应急处置能力，完善安全生产管理机制和责任体系，落实政府、行业监管和企业主体责任，全力抓好安全生产。

　　七、更大力度抓依法行政，加快建设法治政府

　　守法是政府“第一形象”、是施政“第一生命”。各级政府要牢固树立依法行政思维，提高科学决策水平，坚持公正执法，推进政务公开，切实在法治轨道上开展工作、推动发展。

　　以法治促善政，严格规范权力。强化法治思维，改进法治方式，坚持依法用权，确保公权姓公、服务人民。争取成为省首批具有地方立法权的市。加强政府法制工作，推行政府法律顾问制度，完善市、县依法行政工作体系。严格执行决策法定程序，建立重大决策终身责任追究、倒查机制。坚持“逢文必审”、“有错必纠”，加强行政机关内部重大决策合法性审查。落实行政执法全程记录和裁量权基准制度，坚持有法必依、执法必严、违法必究，让群众感受到公平正义的法治阳光。

　　以法治促勤政，依法全面履职。强化行政效能监察，加大行政问责、工作追责力度；强化政府工作督查督办，确保政令畅通。全体公务员尤其是领导干部要讲政治、懂规矩、守纪律，坚持法定职责必须为，法无授权不可为，在干净中干事，在守法中担当；习惯在严格遵章守纪中做好工作，学会在严格自律中担当责任，适应在严格约束下干事创业，创造人民满意的发展实绩！

　　以法治促廉政，强化制约监督。自觉接受人大依法监督和政协民主监督，全面落实人大决议决定，认真办理建议提案。改革公共资源交易体制，把政府采购、土地出让、产权交易、工程招投标纳入公共资源交易平台。推进财政资金、国有资产、政府投资项目和领导干部经济责任审计全覆盖。坚持公开为原则、不公开为例外，全面推进政务公开，重点公开财政预算、公共资源配置、重大建设项目批准实施、社会公益事业建设等政府信息。

　　认真履行党委（党组）主体责任，扎实推进政府系统党风廉政建设和反腐败工作。全面落实中央“八项规定”，持之以恒反“四风”。严格落实省委第七巡视组反馈意见，严肃认真抓好整改工作。坚持有腐必反、有贪必肃，依法依纪严肃处理贪腐行为。严肃财经纪律，加强“三公”经费管理，真正用好公权、管好公款、当好公仆，努力让组织放心、人民满意！

　　各位代表，从经济社会发展全局出发，市政府在全面落实各项任务基础上，今年突出抓好以下五项工作：一是全力办好第14届省运会，进一步展示湛江新形象，彰显湛江新活力，提升湛江美誉度；二是全力保障湛江钢铁基地钢前系统建成投产，实现湛江工业历史性跨越；三是建成保税物流中心（B型），打造对外开放新平台；四是推动全市87所乡镇卫生院纳入县级公立医院管理，完成县镇医院一体化改革；五是完成“十三五”规划编制，突出规划一批可以落地的重点项目、可以实施的重点工程、可以办成的重点实事，还有一批能够推行的改革政策和创新举措，为打造湛江发展升级版，全面建成小康社会绘就蓝图！

　　各位代表！两千年前，我们的先辈从徐闻古港出发，开启了举世闻名的古代海上丝绸之路。30年前，湛江成为国家首批沿海对外开放城市。30年来，沐浴改革开放的春风，湛江迈进发展新时代。今天，湛江已经成为国家“一带一路”海上合作战略支点城市。站在新起点，开启新征程，我们完全有信心、有条件、有能力适应新常态，抢抓新机遇，实现新发展，开创新未来，续写更加辉煌的新篇章！
